# Study protocol and preliminary results from a mono-centric cohort within a trial testing stereotactic body radiotherapy and abiraterone (ARTO-NCT03449719)

**DOI:** 10.1007/s11547-022-01511-7

**Published:** 2022-06-28

**Authors:** Giulio Francolini, Beatrice Detti, Vanessa Di Cataldo, Pietro Garlatti, Michele Aquilano, Andrea Allegra, Sara Lucidi, Cecilia Cerbai, Lucia Pia Ciccone, Viola Salvestrini, Giulia Stocchi, Barbara Guerrieri, Luca Visani, Mauro Loi, Isacco Desideri, Monica Mangoni, Icro Meattini, Lorenzo Livi

**Affiliations:** 1 Radiation Oncology Unit, Azienda Ospedaliera Universitaria Careggi, University of Florence, Largo Brambilla 3, 50139 Florence, Italy; 2 CyberKnife Center, Istituto Fiorentino di Cura ed Assistenza, Florence, Italy; 3 Department of Experimental and Clinical Biomedical Sciences “M. Serio”, University of Florence, Florence, Italy

**Keywords:** Abiraterone, mCRPC, Oligometastatis, SBRT, Protocol

## Abstract

**Background:**

ARTO trial was designed to evaluate the difference in terms of outcomes between patients affected by oligo metastatic castrate resistant prostate cancer (mCRPC) treated with Abiraterone acetate and randomized to receive or not SBRT on all sites of disease. Here, we present a preliminary analysis conducted on patients enrolled at promoting institution.

**Objective:**

To present a preliminary overview about population features, clinical outcomes, adverse events, quality of life and explorative translational research.

**Design, setting, and participants:**

ARTO (NCT03449719) is a phase II trial including patients affected by oligo mCRPC, randomized to receive standard of care (GnRH agonist or antagonist plus abiraterone acetate 1000 mg and oral prednisone 10 mg daily) with or without SBRT on all metastatic sites of disease. All subjects have < 3 bone or nodal metastases. All patients are treated in I line mCRPC setting, no previous lines of treatment for mCRPC are allowed.

**Outcome measurements and statistical analysis:**

Data about a mono-centric cohort of 42 patients enrolled are presented in the current analysis, with focus on baseline population features, PSA drop at 3 months, biochemical response, and quality of life outcomes. Descriptive statistics regarding translational research are also presented.

**Results and limitation:**

Significant difference in terms of PSA drop at three months was not detected (*p* = 0.68). Biochemical response (PSA reduction > 50%) was reported in 73.7 versus 76.5% of patients in control vs SBRT arm, respectively (*p* = 0.84). All patients are alive. Progression occurred in 1 versus 0 patients in the control versus SBRT arm, respectively. After 3 months, an average decrease of 13 points in terms of Global Health Score was reported for the overall population. However, complete recovery was noticed at 6 months. Circulating tumor cells detection rate was 40%.

**Conclusions:**

SBRT + Abiraterone treatment was safe and well tolerated, non-significant trend in terms of PSA drop and biochemical response at 3 months was detected in SBRT arm. Interestingly, CTCs detection in this selected cohort of oligo-mCRPC was lower if compared to historical data of unselected mCRPC patients.

## Background and overall rationale for the study

Prostate cancer (PCa) is one of the most common malignancies and main causes of cancer death in Western countries. Metastatic castration resistant prostate cancer (mCRPC), defined by tumor growth despite a testosterone level of less than 50 ng per deciliter (1.7 nmol per liter) [1], causes approximately 258,400 deaths annually worldwide [2]. Various treatment options for mCRPC have been developed, first the use of Docetaxel in this setting [3], followed by introduction of Androgen receptor targeted agents (ARTA), Enzalutamide and Abiraterone, in pre-docetaxel scenario [4, 5]. All these treatment options showed to significantly improve overall survival if compared to placebo or treatment options previously available (e.g. mitoxantrone), and currently constitute the cornerstone of systemic therapy for mCRPC patients. However, since the introduction of highly sensitive imaging techniques, a new clinical entity of metastatic patients with a limited number of lesions has been defined: oligometastatic patients. This subgroup of patients was first described by Hellman and Weichselbaum in 1995 as an intermediate state between local and widespread metastatic dissemination [6]. In this particular setting, Stereotactic Body Radiotherapy (SBRT) or other local therapies on active lesions have been suggested as possible salvage treatment, both in hormone sensitive [7] and mCRPC setting [8]. Recently, a consensus statement was reached within Italian Association of Radiotherapy and Clinical Oncology (AIRO), aimed to properly define which patients should be defined oligometastatic, standardizing the clinical role of ablative radiotherapy in oligometastatic prostate cancer [9].

The panel agreed that oligometastatic prostate cancer should be defined as ≤ 3 synchronous metastases (bone and/or lymph nodes), and analyzed a particular clinical scenario involving patients affected by oligometastatic CRPC as its first occurrence (e.g. patients developing castration resistance with a number of lesions compatible with oligometastatic definition). Expert agreed that two scenarios are possible in oligometastatic CRPC: administer SBRT with Androgen Deprivation Therapy (ADT) in order to delay ARTA or add SBRT on all oligometastatic sites to ARTA in order to improve disease control. However, ARTA delay could be detrimental considering the known survival benefit of these agents established in randomized trials. Conversely, no safety concern was raised against SBRT to ARTA addition, given the results from patients treated with abiraterone acetate (AA) and concomitant radiotherapy within the pivotal trial COU-AA 301 [10]. Even so, the benefit of the addition of SBRT to ARTA is currently unknown. For this reason, ARTO trial was designed to evaluate the difference in terms of outcomes between patients affected by oligo mCRPC treated with AA and randomized to receive or not SBRT on all sites of disease. Planned accrual sample for this trial is 174 patients. Here, we present a preliminary analysis concerning clinical outcomes, safety of the combination and an explorative overview about translational research conducted within the trial.

## Materials and methods

**Fig. 1:**
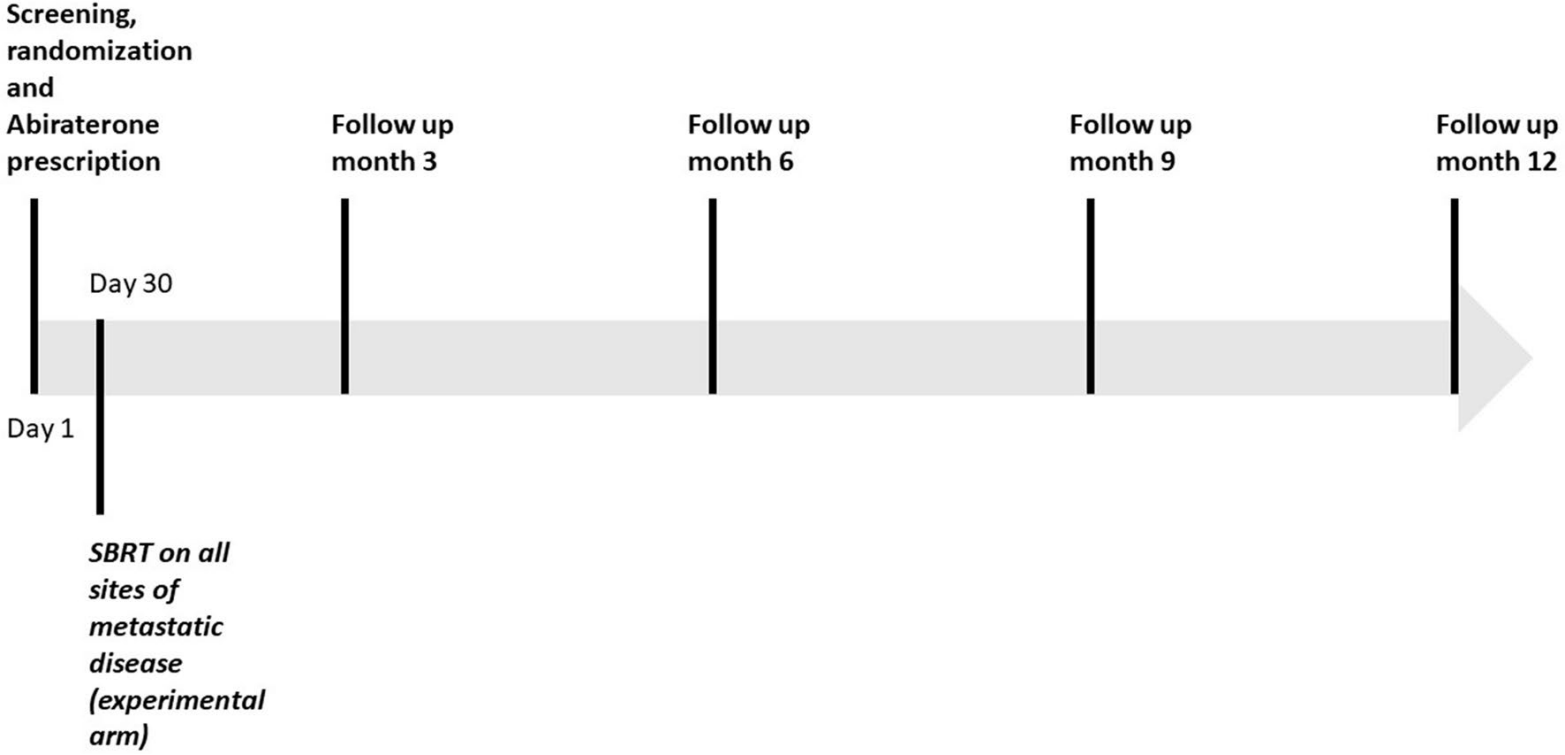
Overview of study design

ARTO (NCT03449719) is a phase II trial including patients affected by oligo mCRPC, randomized to receive standard of care (GnRH agonist or antagonist plus abiraterone acetate 1000 mg and oral prednisone 10 mg daily) with or without SBRT on all metastatic sites of disease. All included subjects have ≤ 3 bone or nodal metastases, patients in whom visceral disease was detected are excluded from the trial. All patients are treated in I line mCRPC setting, no previous lines of treatment for mCRPC are allowed. Primary endpoint of the trial is difference in PSA response rate between the experimental arm (AA + SBRT) and control arm (AA). PSA response is defined as a post-treatment decrease > 50% from baseline measured within 6 months. Baseline assessment of disease is performed with Positron Emission Tomography/Computed Tomography (PET/CT), or Bone Scan and contrast enhanced CT performed within 45 days before randomization. SBRT is delivered in 1–5 fractions, doses and fractionation are decided by treating physician provided that a BED_3_ ≥ 100 is administered. Normal tissue constraints recommended are in accordance to AAPM Task Group 101 [11]. To avoid any influence of SBRT timing on biochemical response, radiotherapy is always started after 30 (± 3) days after systemic treatment beginning. Assessment of PSA, blood test and QoL are performed every 3 and up to 12 months from the start of abiraterone therapy. After the end of study follow up, patients alive and free from progression will continue standard of care treatment. Here, we present a preliminary overview about population features, clinical outcomes, adverse events, quality of life (QoL) and explorative translational research of patients enrolled at trials promoting institution. Principal study procedure and overview of study design are summarized in Table 1 and Fig. 1, respectively. Protocol was approved by Ethical Committee Area Vasta Centro (Approval n. 12855_spe, 09/10/2018).

**Table 1:** Summary of study procedures

1	Inclusion criteria	Exclusion criteria	Demographics, baseline disease features	ECOG score	BPI SF—EORTC QLQ-C30	Complete blood test PSA and testosterone	Baseline staging within 45 days from randomization
Baseline	*x*	*x*	*x*	*x*	*x*	*x*	*x*
Follow up visit (months 3,6,9,12)				*x*	*x*	*x*	*

^*^Same radiological exams used at baseline will be repeated in case of suspected clinical or biochemical progression. Furthermore, in baseline evaluation as well as in case of disease progression, the use of additional diagnostic exams (such as magnetic resonance imaging, MRI) is allowed according to clinical judgement

### Statistical considerations and sample size

Patients are randomly assigned in a 1:1 ratio to both treatment and stratified by random permuted blocks for Centre, Performance status (0–1 vs. > 1) and number of metastases (1 vs. > 1). Randomization is performed the same day of the baseline evaluation (± 3 days). Assuming that the proportion of patients achieving a ≥ 50% PSA level decline in control arm is equal to 62% [12, 13], with a 5% type 1 error rate and a power of 80%, a total of 156 patients are required to show an absolute improvement in proportion of patients in the experimental arm of + 21%. Considering a 10% rate of drop out during follow-up, the final sample size needed is 174 patients (87 for each arm). For the current analysis, descriptive statistics were used to describe population features. Mann–Whitney test was used to compare continuous variables, while chi square test was performed to compare categorical variables.

## Results

### Population

Data from 42 patients enrolled at the promoting institution are available at 1st April 2021, 23 and 19 in the control and treatment arm, respectively. Baseline population features are summarized in Table 2. All patients were treated with standard ADT in metastatic castrate sensitive status, before CRPC occurrence. No differences in terms of median age, median PSA at mCRPC diagnosis, proportion of patients with > 1 distant metastases or ISUP pattern ≥ 3 were detected between treatment arms. Seventy-one percent of the analyzed population underwent Choline PET/CT staging at mCRPC diagnosis.

**Table 2:** Baseline population features

	*n*	*p*
Study arm		
Control	23 (54.8%)	
Treatment	19 (45.2%)	
Median age		
Control	69 (IQR 65–76.2)	0.23
Treatment	73 (IQR 66–78)	
Multiple metastatic sites		
Control	17 (73.9%)	0.07
Treatment	9 (47.4%)	
Baseline ISUP pattern ≥ 3		
Control	17 (73.9%)	0.15
Treatment	10 (52.6%)	
Median PSA at mCRPC occurrence		
Control	2.55 (IQR 1.2–6.11)	0.76
Treatment	2, 91 (IQR 1.6–6.6)	
Staging at mCRPC occurrence		
Choline PET/CT	30 (71.4%)	
Bone scan/CT	1(2.4%)	
PSMA PET/CT	11(26.2%)	

*mCRPC* metastatic castration resistant prostate cancer, *PET/CT* positron emission tomography/computed tomography, *PSMA* prostate specific membrane antigen

### Clinical outcome

At the time of the current analysis, complete data at 3 months after treatment start were available for 36 out of 42 patients, 19 and 17 in the control and treatment arm, respectively. Thirty metastatic lesions were treated in the SBRT arm (7, 8 and 2 patients were treated with LINAC-based intensity modulated RT, Cyberknife(*R*) robotic technique and helical Tomotherapy(*R*), respectively). All patients were treated at trial promoting institution. Significant difference in terms of PSA drop at three months was not detected (*p* = 0.68). Biochemical response (PSA reduction > 50%) was reported in 73.7 versus 76.5% of patients in control vs SBRT arm, respectively (*p* = 0.84). All patients are alive. Progression occurred in 1 versus 0 patients in the control versus SBRT arm, respectively. No adverse events occurred in both arms of treatment. Preliminary results of this cohort were presented at European Multidisciplinary Congress on Urological Cancers (EMUC) in 2020 [14].

### Quality of life

A preliminary analysis about QoL was available for 29 out of 42 patients (16 and 13 in the control and treatment arm, respectively). QoL evaluation by EORTC QLQ-C30 was performed every 3 months after randomization. After three months, an average decrease of 13 points in terms of Global Health Score (GHS) was reported for the overall population. Difference between average values of decrease reported in control and treatment arm (11 vs. 16 points, respectively) did not exceed minimal clinically important difference (MCID). However, complete recovery in terms of GHS was noticed at 6 months. In terms of physical, role, emotional, cognitive and social functioning, reported values were stable if compared to baseline at three and 6 months. A significant influence of SBRT was reported only for social functioning, where a 10 points difference (higher than MCID of 8.4) between control and treatment arm at three months was detected. Complete recovery at 6 months was reported.

### Translational research

An exploratory analysis of androgen receptor splice variants (ARV7/ARFL) Prostate Specific Antigen (PSA) and Prostate Specific Membrane Antigen (PSMA) expression on Circulating Tumor Cells (CTCs) detected in a preliminary cohort of 31 out of 42 patients was presented at ASCO GU 2021. Baseline blood samples to detect CTCs and evaluate their ARV7, ARFL, PSA and PSMA expression were taken before AA treatment start. Results showed a CTC detection rate of 40% of them. Considering only patients in whom circulating tumor cells were detected, 75% did not express ARV7 or AR full length splice variants, while all patients expressed PSMA and 75% of patients had PSA positive CTCs [15].

## Discussion

ARTO trial (NCT03449719) is aimed to assess the benefit of adding SBRT to ARTA in a population of oligometastatic prostate cancer patients. Early results from this trial showed that SBRT + Abiraterone treatment was safe and well tolerated in the experimental cohort, non-significant trend in terms of PSA drop and biochemical response at 3 months was detected in SBRT arm. No increase in terms of adverse events or quality of life impairment if compared to Abiraterone treatment was reported. Interestingly, CTCs detection in this selected cohort of oligo-mCRPC was lower if compared to historical data of unselected mCRPC patients [16]. Final results from ARTO trial will help to explore benefit yielded by SBRT addition to Abiraterone therapy in I line oligo-metastatic CRPC patients and influence current opinion of Italian radiation oncologist in management of oligometastatic prostate cancer patients [17, 18]. Of course, it is very difficult to provide insights for future implementation of SBRT in the rapidly changing landscape of hormonal treatment in metastatic prostate cancer patients, after the introduction of ARTA in non-metastatic and hormone sensitive settings. However, additive effect of SBRT to ARTA could be translated to different scenarios, provided that local approach should probably be reserved to early disease stages, through upfront detection of patients with indolent disease. Currently, outcomes of this strategy have been tested mainly in retrospective analyses [8]. Prospective data from the SABR-COMET may significantly change the landscape of this scenario, showing significant improvement in overall survival after addition of SBRT to standard of care if compared to standard of care alone [19]. However, SABR-COMET trial included a mixed population of metastatic cancer patients, conversely, ARTO trial will explore the addition of SBRT to standard of care treatment in a well-selected population of mCRPC patients, all treated with I line ARTA. Thus, results from this trial, together with other ongoing trials testing the benefit of SBRT addition to systemic therapy in CRPC (NCT03556904 and NCT04319783 trials). Of course, use of ARTA for treatment of prostate cancer is rapidly evolving due to results of trials testing the benefit of this class of drugs in high risk metastatic hormone sensitive prostate cancer [20, 21] in non-metastatic CRPC [22] and also in local or locally advanced prostate cancer [23], shifting the addition of Abiraterone, Apalutamide or Enzalutamide in earlier phases of disease. For this reason, it will be useful to develop integrated treatment strategies between SBRT and systemic treatment in this complex scenario [24, 25]. One of the major criticism of current clinical practice is the lack of consensus about staging methods in mCRPC (e.g. whether a conventional staging, a choline or PSMA PET/CT should be used in this setting). Interestingly, only 1 out of 42 patients of the present cohort was staged with conventional imaging, and 97.6% of patients were staged with Choline or PSMA PET/CT scan. Thus, there is no risk of understaging in the analyzed population. According to American Society for Clinical Oncology (ASCO) guidelines, conventional imaging can be used for initial evaluation of PSA progression and should be continued to facilitate changes/comparisons and serially to assess for development of radiographic progression [26]. For this reason, a formal exclusion criterion for conventional staging was not implemented in the protocol. Nonetheless, due to current clinical practice, vast majority of enrolled patients will continue to be staged at baseline with Choline or PSMA PET/CT. Moreover, it would be really interesting to test whether an earlier re-staging in CRPC patients (e.g. Choline vs PSMA imaging) will results in earlier treatment and improved efficacy in this setting*.* Given the actual debate about use of PSMA imaging in CRPC setting, protocol will not be amended to provide PSMA staging in all new enrolled patients. Imaging methods will remain according to local clinician choice and future analysis will be explorative only. Regarding the trial design, a control arm including SBRT + ADT without ARTA was not implemented in ARTO trial. Indeed, overall survival benefit of ARTA in mCRPC setting was already shown before the design of ARTO Trial, after results from COU-AA-302 and PREVAIL trials [4, 5]*.* For this reason, the hypothesis of using SBRT + ADT to spare and delay ARTA would probably result in undertreatment of these patients, and Italian Association of Radiotherapy and Clinical Oncology (AIRO) did not endorsed this treatment strategy [9]*.* Primary endpoint of ARTO trial may seem questionable, but PSA response has been tested as a surrogate endpoint after radical treatment and showed to be a strong prognostic biomarker for biochemical progression-free survival (bPFS), Prostate Cancer specific survival (PCSS), and OS [27]. Thus, results from ARTO trial may establish a substantial evidence of the benefit of this treatment strategy in oligo mCRPC patients treated with ARTA. Moreover, Data about ARV7, ARFL, PSA and PSMA expression will represent an interesting snapshot of biomarker arrangement in this setting.
